# Supplementary material for: In vivo assessment of a delayed release formulation of larazotide acetate indicated for celiac disease using a porcine model
Source: PLoS One. 2021 Apr 12;16(4):e0249179. doi: 10.1371/journal.pone.0249179 (PMC8041193; doi:10.1371/journal.pone.0249179)
Supplement: S1 Text — (PDF) [file pone.0249179.s001.pdf]

In vitro experiment

Chemicals and Reagents

Larazotide analytical reference standard was purchased from Corden pharma. Deionized water was purchased from UPM. Potassium phosphate monobasic, 1N hydrochloric acid, trifluoroacetic acid (TFA) and Tween 80 were purchased from Fisher science. 0.2 N sodium hydroxide was purchased from VWR and acetonitrile (ACN) was purchased from J.T. Baker. Simulated intestinal fluid and gastric fluid were purchased from Ricca chemicals.

Simulated gastric dissolution media (gastric fluid, 0.1% tween 80)

1 L of simulated gastric fluid dissolution media was prepared by mixing 1 L of simulated gastric fluid previously degassed by helium sparging for approximately 5 minutes and 1 mL of tween 80.

Simulated intestinal dissolution media (intestinal fluid, pH 6, 0.1% tween 80)

1 L of simulated intestinal fluid was prepared by mixing 1 L of simulated intestinal fluid previously degassed by helium sparging for approximately 5 minutes and 1 mL of tween 80 and adjusted the pH to 6.0 with 1 N hydrochloric acid.

0.2 N potassium phosphate monobasic

1 L of 0.2 N potassium phosphate monobasic was prepared by dissolving 27.22 g of potassium phosphate monobasic in 1 L of deionized water.

50 mM phosphate buffer (pH7)

1 L of 50 mM phosphate buffer was prepared by mixing 250 mL of 0.2 N potassium phosphate monobasic and 145 mL of 0.2 N sodium hydroxide. It was diluted with deionized water to 1 L.

Instrumentation

Analysis of LA was carried out via High performance liquid chromatography (HPLC). HPLC system consists of 2690 separations module and 2487 dual wavelength absorbance detector (Waters Corporation, Milford, MA) and empower chromatography data system.

Preparation of stock solution

5 mg of LA reference standard was weighed after correction with peptide purity and peptide content by using the equation shown below. 15 mL of 50 mM phosphate buffer (pH=7) was added and sonicated for 5 minutes. It was diluted with 50 mM phosphate buffer (pH=7) to 25 mL. The nominal concentration was 0.2 mg / mL.

Equation of correction for peptide purity and peptide content:

5 mg / {(peptide purity % /100) x (peptide content %) / 100}

Preparation of standard solution (simulated gastric media)

2.0 mL of the stock solution was diluted with simulated gastric media and mixed well. 5 mL of this solution was diluted with 45 mL simulated gastric media to make 0.0002 mg / mL.

Preparation of standard solution (simulated intestinal media)

2.0 mL of the stock solution was diluted with 198 mL simulated intestinal media and mixed well to make 0.002 mg / mL.

Dissolution condition

The dissolution method was performed according to the current UPS dissolution monograph chapter 711[23] under the following conditions. In simulated gastric media, apparatus I baskets with 500 mL 0.1 % tween 80 simulated gastric media was used at 37.0 ± 0.5 ºC. The speed was set at 100 RPM and 5 mL of sample was collected at 30, 60 and 120 minutes respectively. In simulated intestinal media, apparatus I baskets with 500 mL 0.1 % tween 80

simulated intestinal media was used at 37.0 ± 0.5 ºC. The speed was set at 100 RPM and 5 mL of sample was collected at 15, 30, 45, 60, 90, 120 and 180 minutes respectively.

Dissolution test procedure

Six capsules were weighed separately (each capsule included 1 mg LA beads). One capsule was placed into each basket and began the dissolution by using the simulated gastric media. 5 mL sample was collected at each time points described previously. Each sample was filtered via 0.45 µm nylon filter and first 2 mL was discarded. The remaining was injected into HPLC for analysis. The same procedure was taken place by using simulated intestinal media. The appropriate dissolution conditions described previously were used.

HPLC conditions

Chromatographic separation was performed by a gradient elution on the Alltima HP C18 Hi-load 5 µm column (150 mm x 3.0 mm). The mobile phase solvents were 0.1 % TFA in 85 % deionized water and 15 % ACN (A) and 0.1 % TFA in 100 % ACN (B) at a flow rate of 1.0 mL /min for 20 minutes. The gradient program mobile phase conditions were 100 % of A and 0 % of B for the first 6 minutes, then changed linearly to 25 % of A and 75 % of B from 6.1-11 minutes, then immediately back to 100 % of A and 0 % of B from 11.1-20 minutes to re-equilibrate at the initial conditions. The column temperature was 40 °C, the autosampler temperature was maintained at 5 °C. The detector wavelength was 220 nm. The retention time was 3 minute.

Method validation

This method was validated following the FDA Bioanalytical Method Validation Guidelines for Industry [24]. Accuracy, precision, recovery, system precision, linearity, specificity, filter tip interference, matrix effect, and stability were assessed. Full validation was performed using simulated gastric media and simulated intestinal media.

Precision and accuracy

A total of 6 replicates of 1 mg LA capsules were dissolved and collected at 120 minutes sampling time point and tested within one day by first analyst. Accuracy and precision were calculated with the requirement that the mean must be within 15 % of the nominal value and must have a precision not exceeding 15 % coefficient of variation (CV). A second analyst independently analyzed 6 samples using different dissolution system, standards, solutions, column, and instrument on a different day. The % difference between analyst 1 and analyst 2 were evaluated (2-7 %).

Stability

Chemical stability of 1 mg LA standard solution and sample solution were evaluated under refrigerated conditions for at least 72 hours with two intermediate time points. Difference must be within 3.0 % of the freshly prepared standard solution and initial concentration respectively.

System suitability requirements

The blank simulated gastric media was injected to check any interfering peaks. Six injections of Larazotide acetate working standard solution were performed. The CV % of the active name peak area responses should be not more than 5 %. One injection of Larazotide acetate standard was checked. The % recovery must be within 95 % to 105 % of the theoretical value. The % recovery of bracketing standard should be within 95 % to 105 %. The blank simulated intestinal media was injected to check any interfering peaks. Six injections of Larazotide acetate working standard solution were performed. The CV % of the active name peak area responses should be not more than 5 %. One injection of Larazotide acetate standard

was checked. The % recovery must be within 92 % to 108 % of the theoretical value. The % recovery of bracketing standard should be within 92 % to 108 %.

Calculation

Dissolved % of LA was calculated by using the equation described below. Subsequent timepoints are calculated as for Timepoint 3, with the inclusion of the response obtained for the timepoints, in the appropriate sections of the calculations.

Timepoint 1

Dissolve % = (R_{sam1}/R_{std}) x V x C_{std} x 1/L x 100 = R_{sam1}/R_{std} x 500 x C_{std} x 1/L x100

Timepoint 2

Dissolved % = $[(R_{sam2}/R_{std} \text{ x } (v\text{-}TPV_{(n-1)})) + (R_{sam1}/R_{std} \text{ x } PV)] \text{ x } C_{std} \text{ x} 1/L \text{ x} 100$

$= [(R_{sam2}/R_{std} \text{ x } 495) + (R_{sam1}/R_{std}) \text{ x } 5] \text{ x } C_{std} \text{ x } 1/L \text{ x} 100$

Timepoint 3

Dissolved % = $\{[(R_{sam3}/R_{std} \text{ x } (v\text{-}TPV_{(n-2)})] + [(R_{sam1} + R_{sam2})/R_{std} \text{ x } PV]\} \text{ x } C_{std} \text{ x } 1/L \text{ x } 100$

$= [(R_{sam3}/R_{std} \text{ x } 490) + \{(R_{sam1} + R_{sam2}) / R_{std} \text{ x } 5\}] \text{x } C_{std} \text{ x } 1/L \text{ x } 100$

R_{sam} : Peak area response of Larazotide acetate obtained from the sample chromatogram

R_{std} : Average peak area responses of Larazotide acetate obtained from all the system suitability injections

C_{std} : Concentration, in mg/mL, of Larazotide acetate standard solution

V : Initial volume of dissolution media (500 mL)

PV : Sample pull volume, 5 mL

TPV : Total sample pull volume, 5 x (n-1) mL (time point n=1, 2, 3…)

100 : Conversion of percentage

L : Labeled amount
