# Supplementary figures and images for: In vivo assessment of a delayed release formulation of larazotide acetate indicated for celiac disease using a porcine model
Source: PLoS One. 2021 Apr 12;16(4):e0249179. doi: 10.1371/journal.pone.0249179 (PMC8041193; doi:10.1371/journal.pone.0249179)

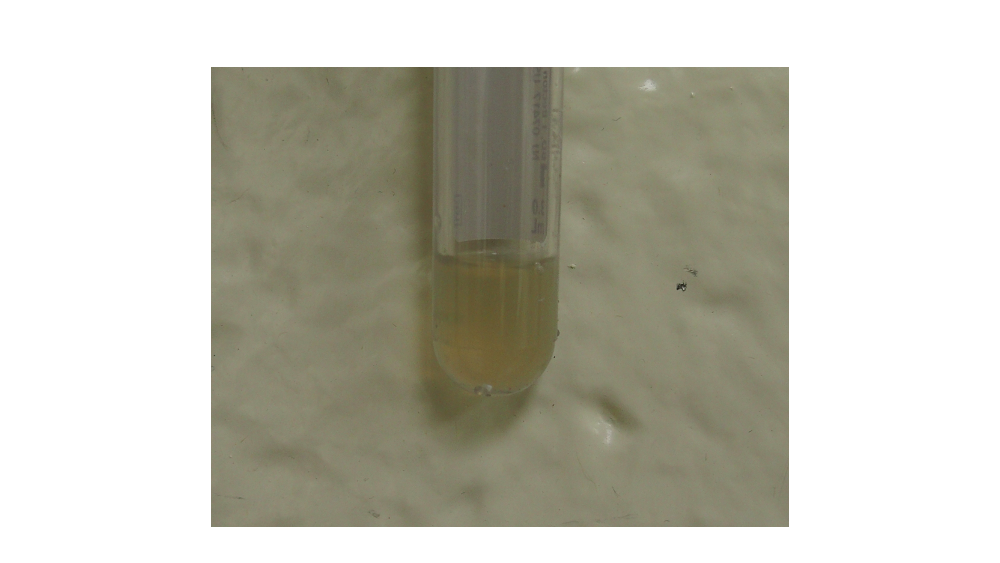

Supplement: S1 Fig — (TIF) [file pone.0249179.s002.tif]

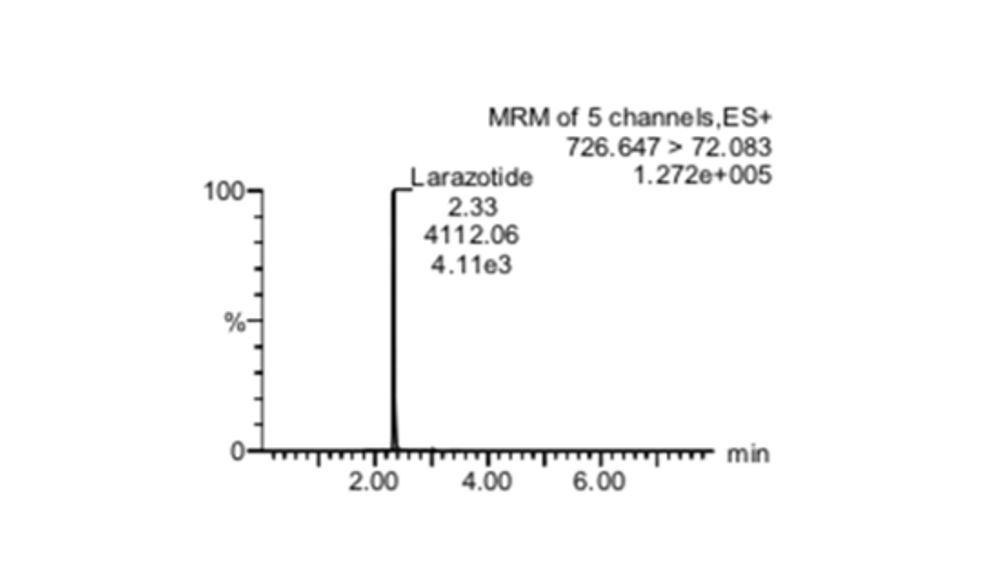

Supplement: S2 Fig — (TIF) [file pone.0249179.s003.tif]

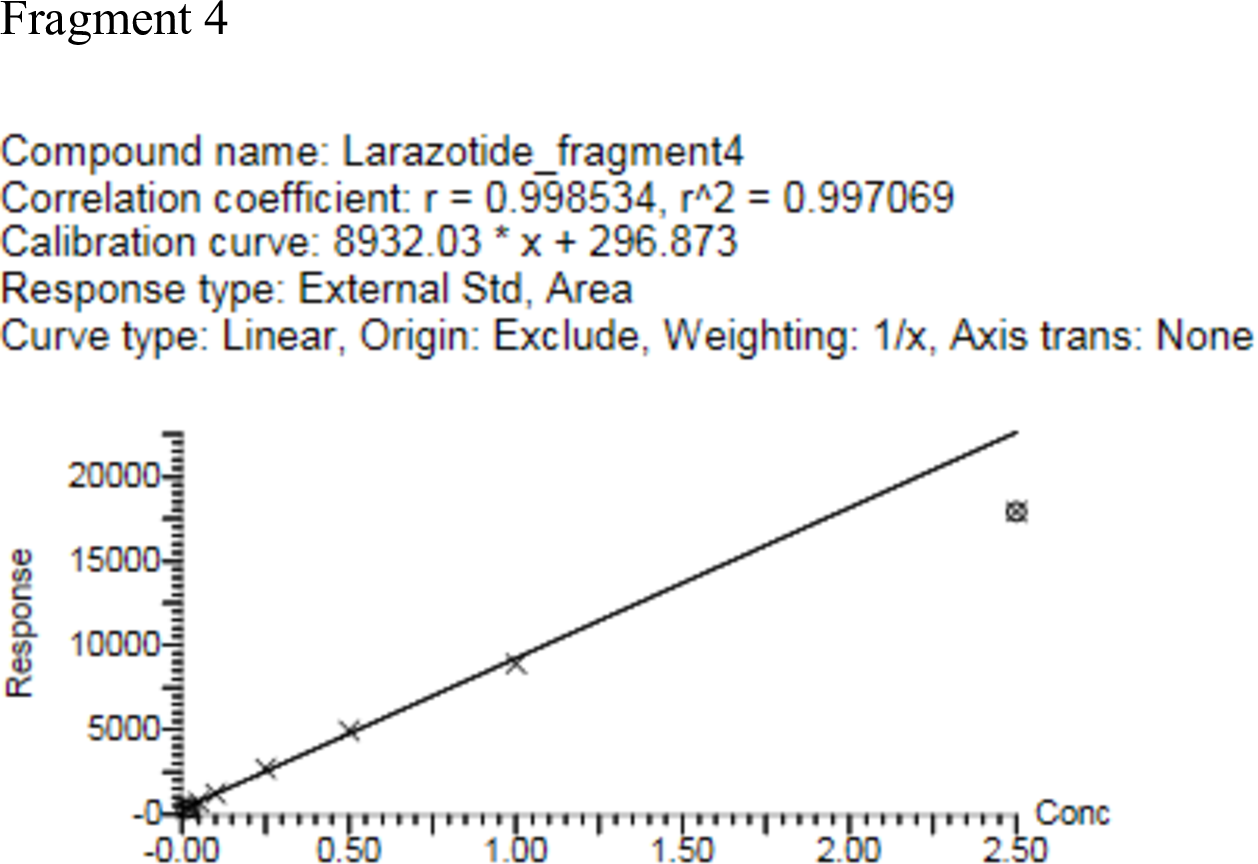

Supplement: S3 Fig — (TIF) [file pone.0249179.s004.tif]
