# Supplementary material for: In vivo assessment of a delayed release formulation of larazotide acetate indicated for celiac disease using a porcine model
Source: PLoS One. 2021 Apr 12;16(4):e0249179. doi: 10.1371/journal.pone.0249179 (PMC8041193; doi:10.1371/journal.pone.0249179)
Supplement: S1 Table — (PDF) [file pone.0249179.s005.pdf]

ID	RT (mins)	Major precursor mass (m/z)	Daughter mass(m/z)	Precursor form	Structure
LA	2.33	726.64	72.08	(M+H)+	H-Gly-Gly-Val-Leu-Val-Gln-Pro-Gly-OH
Fragment 1	2.32	669.54	86.14	(M+H)+	H-Gly-Val-Leu-Val-Gln-Pro-Gly-OH
Fragment 2	2.23	612.58	72.1	(M+H)+	H-Val-Leu-Val-Gln-Pro-Gly-OH
Fragment 3	2.43	669.54	72.1	(M+H)+	H-Gly-Gly-Val-Leu-Val-Gln-Pro-OH
Fragment 4	2.35	555.64	72.1	(M+H)+	H -Val-Leu-Val-Gln-Pro-OH
