# Supplementary material for: In vivo assessment of a delayed release formulation of larazotide acetate indicated for celiac disease using a porcine model
Source: PLoS One. 2021 Apr 12;16(4):e0249179. doi: 10.1371/journal.pone.0249179 (PMC8041193; doi:10.1371/journal.pone.0249179)
Supplement: S2 Table — (PDF) [file pone.0249179.s006.pdf]

Analytical method suitability study data

Intraday precision and accuracy (n=6 replicates within one day) for pig intestinal fluid samples. Six pig intestinal fluid samples were spiked with larazotide, fragment 1, 2, 3 and 4 at concentration of larazotide (0.21, 1.05 and 2.8 µM), fragment 1 (0.45, 1.13 and 3 µM), fragment 2 (0.48, 1.2 and 3.2 µM), fragment 3 (0.45, 1.13 and 3 µM) and fragment 4 (0.51, 1.28 and 3.4 µM).

Analyte	Spiking	Average	SD	Recovery	CV
Larazotide	concentration (µM)	estimate concentration (µM)	(standard deviation)	(%)	(coefficient of variation %)
	0.21	0.23	0.008	107.32	3.73
	1.05	1.13	0.025	107.78	2.25
	2.8	2.89	0.045	102.45	1.51

Analyte	Spiking	Average	SD	Recovery	CV
Fragment 1	concentration (µM)	estimate concentration (µM)	(standard deviation)	(%)	(coefficient of variation %)
	0.45	0.43	0.014	94.78	4.89
	1.13	1.09	0.019	97.28	2.61
	3	2.8	0.161	93.42	8.6

Analyte	Spiking	Average	SD	Recovery	CV
Fragment 2	concentration (µM)	estimate concentration (µM)	(standard deviation)	(%)	(coefficient of variation %)
	0.48	0.46	0.043	96.05	14.85
	1.2	1.13	0.074	94.55	10.39
	3.2	2.88	0.165	90.12	9.14

Analyte	Spiking	Average	SD	Recovery	CV
Fragment 3	concentration (µM)	estimate concentration (µM)	(standard deviation)	(%)	(coefficient of variation %)
	0.45	0.42	0.012	93.72	4.33
	1.13	1.03	0.026	91.93	3.77
	3	2.74	0.035	91.32	2.74

Analyte	Spiking	Average	SD	Recovery	CV
Fragment 4	concentration (µM)	estimate concentration (µM)	(standard deviation)	(%)	(coefficient of variation %)
	0.51	0.5	0.017	98.9	5.58

	1.28	1.33	0.015	104.55	1.88
	3.4	3.54	0.018	104.33	0.88
