# Supplementary material for: In vivo assessment of a delayed release formulation of larazotide acetate indicated for celiac disease using a porcine model
Source: PLoS One. 2021 Apr 12;16(4):e0249179. doi: 10.1371/journal.pone.0249179 (PMC8041193; doi:10.1371/journal.pone.0249179)
Supplement: S3 Table — (PDF) [file pone.0249179.s007.pdf]

Collected intestinal fluid (μL) via ultrafiltration probe

administered drug : human clinical formulation

< distal duodenum >

case number						
	Time (hr)	0h	1h	2h	3h	4h
1	Intestinal fluid (μL)	2472	436	868	578	487
	Time (hr)	0h	1h	2h	3h	4h
2	Intestinal fluid (μL)	2699	970	903	410	349
	Time (hr)	0h	1h	2h	3h	4h
3	Intestinal fluid (μL)	2577	407	735	558	626
	Average (μL)	2583	604	835	515	487
	Stndard deviation	93	259	72	75	113

< proximal jejunum >

case number						
	Time (hr)	0h	1h	2h	3h	4h
1	Intestinal fluid (μL)	2988	751	504	916	793
	Time (hr)	0h	1h	2h	3h	4h
2	Intestinal fluid (μL)	2588	623	534	497	211
	Time (hr)	0h	1h	2h	3h	4h
3	Intestinal fluid (μL)	1516	787	686	883	664
	Average (μL)	2364	720	575	765	556
	Stndard deviation	621	70	80	190	250

administered drug : placebo

< distal duodenum >

case number						
	Time (hr)	0h	1h	2h	3h	4h
1	Intestinal fluid (μL)	606	982	874	497	893
	Time (hr)	0h	1h	2h	3h	4h
2	Intestinal fluid (μL)	2839	784	554	436	492
	Time (hr)	0h	1h	2h	3h	4h
3	Intestinal fluid (μL)	2100	715	555	565	610
	Average (μL)	1848	827	661	499	665
	Stndard deviation	929	113	151	53	168

< proximal jejunum >

case number						
	Time (hr)	0h	1h	2h	3h	4h
1	Intestinal fluid (μL)	1234	1010	647	519	409
	Time (hr)	0h	1h	2h	3h	4h
2	Intestinal fluid (μL)	1918	1082	811	727	768
	Time (hr)	0h	1h	2h	3h	4h
3	Intestinal fluid (μL)	2100	738	517	584	613
	Average (μL)	1751	943	658	610	597
	Stndard deviation	373	148	120	87	147
