# Supplementary material for: In vivo assessment of a delayed release formulation of larazotide acetate indicated for celiac disease using a porcine model
Source: PLoS One. 2021 Apr 12;16(4):e0249179. doi: 10.1371/journal.pone.0249179 (PMC8041193; doi:10.1371/journal.pone.0249179)
Supplement: S4 Table — (PDF) [file pone.0249179.s008.pdf]

Concentration data of LA (μM) in the intestinal samples (n=3)

administered drug : human clinical formulation

< distal duodenum >

case number						
	Time (hr)	0h	1h	2h	3h	4h
1	LA concentration (μM)	0.00	0.32	0.14	0.03	0.03
	Time (hr)	0h	1h	2h	3h	4h
2	LA concentration (μM)	0.00	0.98	0.21	0.09	0.02
	Time (hr)	0h	1h	2h	3h	4h
3	LA concentration (μM)	0.00	0.93	0.47	0.25	0.03
	Average LA (μM)	0.00	0.74	0.27	0.12	0.03
	Standard deviation	0.00	0.30	0.14	0.09	0.01

< proximal jejunum>

case number						
	Time (hr)	0h	1h	2h	3h	4h
1	LA concentration (μM)	0.00	0.65	0.13	0.04	0.00
	Time (hr)	0h	1h	2h	3h	4h
2	LA concentration(μM)	0.00	1.76	0.18	0.00	0.09
	Time (hr)	0h	1h	2h	3h	4h
3	LA concentration(μM)	0.00	0.88	0.43	0.22	0.00
	Average LA (μM)	0.00	1.10	0.25	0.09	0.03
	Standard deviation	0.00	0.48	0.13	0.10	0.04

administered drug : placebo that does not include LA

< distal duodenum >

case number						
	Time (hr)	0h	1h	2h	3h	4h
1	LA concentration (μM)	0.00	0.00	0.00	0.00	0.00
	Time (hr)	0h	1h	2h	3h	4h
2	LA concentration(μM)	0.00	0.00	0.00	0.00	0.00
	Time (hr)	0h	1h	2h	3h	4h
3	LA concentration(μM)	0.00	0.00	0.00	0.00	0.00
	Average LA (μM)	0.00	0.00	0.00	0.00	0.00
	Standard deviation	0.00	0.00	0.00	0.00	0.00

< proximal jejunum >

case number						
	Time (hr)	0h	1h	2h	3h	4h
1	LA concentration (μM)	0.00	0.00	0.00	0.00	0.00
	Time (hr)	0h	1h	2h	3h	4h
2	LA concentration(μM)	0.00	0.00	0.00	0.00	0.00
	Time (hr)	0h	1h	2h	3h	4h
3	LA concentration(μM)	0.00	0.00	0.00	0.00	0.00
	Average LA (μM)	0.00	0.00	0.00	0.00	0.00
	Standard deviation	0.00	0.00	0.00	0.00	0.00
