# Supplementary material for: In vivo assessment of a delayed release formulation of larazotide acetate indicated for celiac disease using a porcine model
Source: PLoS One. 2021 Apr 12;16(4):e0249179. doi: 10.1371/journal.pone.0249179 (PMC8041193; doi:10.1371/journal.pone.0249179)
Supplement: Attachment — Submitted filename: Enomoto et al., Response to reviewers 11 30 2020.docx [file pone.0249179.s009.docx]

Point-by-point Rebuttal PONE-D-20-20338
“In-vivo assessment of a delayed release formulation of larazotide acetate indicated for celiac disease using a porcine model”

We thank both reviewers for their time and constructive comments to strengthen our manuscript. This document addresses each Reviewer’s concerns. The revised manuscript contains highlighted text for the significant additions to the manuscript and used “track changes” to show deleted text. We look forward to your response.

Sincerely,

Kristen Messenger

2.1. Please provide an amended Funding Statement declaring this commercial affiliation, as well as a statement regarding the Role of Funders in your study. If the funding organization did not play a role in the study design, data collection and analysis, decision to publish, or preparation of the manuscript and only provided financial support in the form of authors' salaries and/or research materials, please review your statements relating to the author contributions, and ensure you have specifically and accurately indicated the role(s) that these authors had in your study. You can update author roles in the Author Contributions section of the online submission form.

*Answer : We have amended this section and included the information in our Cover Letter. This study was funded by Innovate Biopharmaceuticals Inc., which is now 9meters Biopharma. Funding included salary support for authors (KM and AB). RK, JM, and SL were employees of Innovate Biopharmaceuticals and are now either employees or consultants for 9meters. Innovate Biopharmaceuticals (RK, JM, and SL) was involved in the study design, data collection, data analysis, decision to publish, and preparation of the manuscript.*

Please also include the following statement within your amended Funding Statement.

“The funder provided support in the form of salaries for authors [insert relevant initials], but did not have any additional role in the study design, data collection and analysis, decision to publish, or preparation of the manuscript. The specific roles of these authors are articulated in the ‘author contributions’ section.”

If your commercial affiliation did play a role in your study, please state and explain this role within your updated Funding Statement.

*Answer: See above.*

2.2. Please also provide an updated Competing Interests Statement declaring this commercial affiliation along with any other relevant declarations relating to employment, consultancy, patents, products in development, or marketed products, etc.

Within your Competing Interests Statement, please confirm that this commercial affiliation does not alter your adherence to all PLOS ONE policies on sharing data and materials by including the following statement: "This does not alter our adherence to PLOS ONE policies on sharing data and materials.” (as detailed online in our guide for authors http://journals.plos.org/plosone/s/competing-interests). If this adherence statement is not accurate and  there are restrictions on sharing of data and/or materials, please state these. Please note that we cannot proceed with consideration of your article until this information has been declared.

Please include both an updated Funding Statement and Competing Interests Statement in your cover letter. We will change the online submission form on your behalf.

Please know it is PLOS ONE policy for corresponding authors to declare, on behalf of all authors, all potential competing interests for the purposes of transparency. PLOS defines a competing interest as anything that interferes with, or could reasonably be perceived as interfering with, the full and objective presentation, peer review, editorial decision-making, or publication of research or non-research articles submitted to one of the journals. Competing interests can be financial or non-financial, professional, or personal. Competing interests can arise in relationship to an organization or another person. Please follow this link to our website for more details on competing interests: <http://journals.plos.org/plosone/s/competing-interests>

Answer: *Updated and included in the Cover Letter*

3. We note that you have indicated that data from this study are available upon request. PLOS only allows data to be available upon request if there are legal or ethical restrictions on sharing data publicly. For information on unacceptable data access restrictions, please see <http://journals.plos.org/plosone/s/data-availability#loc-unacceptable-data-access-restrictions>.

Answer: *We have provided all the data in Supplementary Files.*

In your revised cover letter, please address the following prompts:

a) If there are ethical or legal restrictions on sharing a de-identified data set, please explain them in detail (e.g., data contain potentially identifying or sensitive patient information) and who has imposed them (e.g., an ethics committee). Please also provide contact information for a data access committee, ethics committee, or other institutional body to which data requests may be sent.

b) If there are no restrictions, please upload the minimal anonymized data set necessary to replicate your study findings as either Supporting Information files or to a stable, public repository and provide us with the relevant URLs, DOIs, or accession numbers. Please see <http://www.bmj.com/content/340/bmj.c181.long> for guidelines on how to de-identify and prepare clinical data for publication. For a list of acceptable repositories, please see <http://journals.plos.org/plosone/s/data-availability#loc-recommended-repositories>.

We will update your Data Availability statement on your behalf to reflect the information you provide.

**Reviewer 1.**

1. The abstract seems too descriptive; the authors are advised to make the abstract short and concise.

*Answer: Thank you very much for your advice. We shortened the abstract.*

2. The term “novel’ for the porcine model used is ambiguous, the literature review does show other porcine models for this type of study. The authors need to show ceratin validation of this “novel” method developed or used in this study. This is “novel” accounted if the authors have independently published this porcine protocol and got approval from regulatory agencies or cited by fellow researchers after using this reported method. A clear justification is requested. If the authors want to report only the novel porcine model and analytical method developed then the title has to be changed suitably.

*Answer: We have removed “novel” from the model description and title.*

3. The authors are advised not to define what methodologies were followed or developed in the abstract making it verbose, instead, those can be reported as results are highly recommended. Mere mentioning about first development or use of any method in abstract does not increase the scientific quality or acceptance.

*Answer: The abstract has been revised as suggested.*

4. Including abstract the entire manuscript is recommended for extensive English grammar revision, making sentences formal and direct and not either way.

*Answer: All has been revised.*

5. From the abstract, the readers would get confused, whether the author's would like to elevate the porcine model or absorbance of delayed LA formulation in. intestine- contradicts with aim defined in the abstract. Kindly rephrase.

*Answer: Abstract has been revised.*

6. The conclusion in the abstract is poorly defined. Kindly rewrite.

*Answer: Revised.*

7. Keywords need to be rechecked.

*Answer: We checked and revised them. Key words are now celiac disease, larazotide acetate, porcine, ultrafiltration*

8. The sentence LA is being studied in phase 3 clinical trials using a clinical formulation designed to reach the target site for treatment of CeD in the small intestine [1]”- the reference cited seems no connection to this sentence. Kindly recheck and cite appropriately.

*Answer: The citation has been updated.*

9. The introduction is poorly constructed. The objective defined at the end again contradicts with title and abstract written. How can we use a non-validated animal model for IVIVC? As per which guidelines the authors have performed the comparison? Kindly cite or remove the phrase. Complete revision is highly recommended.

*Answer: We removed the phrase of in vivo/ in vitro comparison and revised the conclusion, and revised the entire introduction. As you will read in the Discussion, IVIVC could not be performed for this study since only a single formulation was tested and there is no completely dissolved formulation of LA to compare with, although future studies could focus on different formulations and IVIVC could be more useful.*

10. The surgical procedures lack citing the references from where at least the method was inspired or developed.

*Answer: We added a citation on a bovine GI model from which the method was inspired.*

11. The drug formulation lacks to disclose the type of polymers or excipients used to delay or make a trigger release in the duodenum. In-vitro dissolution procedure is not reported or cited. The authors need to carefully address this lacuna.

*Answer: The manuscript has been updated. For the reviewer, the strategy for the manufacturing of the larazotide acetate delayed release capsules involved four layers applied over inert sugar cores. The first layer applied is a drug layer and the second layer applied is a sub coat layer. The third layer applied is an enteric coated layer and the fourth layer is a topcoat layer*.

*Enteric coat layer*

*This layer includes an enteric methacrylate polymer, eudragit, which is well known as an enteric coating polymer that delays the release of the active substance in the acidic surroundings of the stomach. The enteric coat layer enables to release Larazotide at pH level above pH 5 thus allow release in the duodenum as well as jejunum and ileum over the course of about 3 hours when the product exposed to simulated intestinal fluid.*

*The release rate is tested in vitro by an in house dissolution test method simulating the gastric fluid and the intestinal fluid. The development of the dissolution test method was based on the physicochemical in vitro and in vivo characteristics of the product considering the mechanism of release.*

*The in vitro dissolution test for Larazotide is capable of:*

- *discriminating between batches with respect to critical process parameters which may have an impact on the desired bioavailability.*
- *testing for batch to batch consistency of pivotal clinical, bioavailability and routine production batches.*
- *determining stability of the relevant release characteristics of the product over the proposed shelf life and storage conditions.*

*The specification parameters set for dissolution testing are:*

*In simulated gastric fluid:*

- *After 120 minutes not more than 10 % of the labeled amount of Larazotide should dissolve in gastric fluid.*

*In simulated intestinal fluid*

- *Not less than 85 % of the labeled amount of Larazotide should dissolve in 120 minutes.*

12. Authors have failed to mention or cite using or as per which regulatory guidelines the analytical method was developed and validated. If validated why all parameters were disclosed.

*Answer: The method was validated by showing intraday and interday precision and accuracy according to the FDA Bioanalytical Method Validation Guidelines for Industry. We uploaded the method suitability study data to the supplementary file.*

13. Apart from indicating concentration at distal or proximal duodenum by using analytical method, there is no substantial evidence provided by authors on explaining why the formulation could have restricted and had triggered at a specific site. The authors could have mentioned the polymer or excipient used for mucoadhesiveness, formulate into tablet and take x-ray images to locate and see the presence of the beads and then quantify the LA. This seems to be a more justified approach. The authors need to clarify on this.

*Answer: We have now included the in vitro dissolution test developed to serve as a surrogate marker for in vivo behavior and thereby confirm consistent therapeutic performance of batches from routine production. We don’t think that a pig abdominal radiograph would have sufficient resolution to see the beads, although this is a consideration for future studies. We think that measuring the concentration of larazotide acetate directly from different sites in the intestine is important to see larazotide on the surface of the intestinal membrane at the target site in dynamic environment.*

14. There are various contamination and infections borne while using probes. The authors did not mention how they have taken measures to minimize this. Authors need to address this.

*Answer: We performed the routine surgical preparation of the abdomen by using chlorhexidine/alcohol alternating scrubs, preoperative administration of antibiotics (ceftiofur sodium 8.0 mg/kg), used sterile ultrafiltration probes and close observation of the incision daily for signs of infection, including heat, pain, swelling or discharge from the surgical incision. Our lab has extensive experience using these probes and to date we have not had any serious issues with contamination or infections while using the probes as directed.*

15. The study has only two studies- one analytical method to quantify LA and porcine model to collect sampling. The authors have failed to constructively frame the IVIVC. The authors are advised to follow regulatory guidelines while drawing a comparison study.

*Answer: We could not perform the IVIVC because this study did not have the immediate release formulation and/or other multiple formulations for testing and validation of the model. This study design was not enough to compare in vivo data to in vitro data. Thus, we removed the phrase “in vivo data corresponded to in vitro data”.*

**Reviewer 2.**

Introduction

1. Along with the publications [14-17] already cited by the authors, it may be worthwhile to include another reference utilizing a very similar in vivo study for intestinal tract measurements in pigs for a different orally administered drug:
   Yan, L., Xie, S., Chen, D. et al. Pharmacokinetic and pharmacodynamic modeling of cyadox against Clostridium perfringens in swine. Sci Rep 7, 4064 (2017). <https://doi.org/10.1038/s41598-017-03970-9>

*Answer: Thank you very much for your advice. We added this paper to the reference although it is important to note that this reference refers to ex-vivo model.*

2. While the introduction provides a good overview of the need for a minimally invasive, real-time sampling method for in vivo GI profiling of an oral drug, it does not provide a concrete conclusion about the stated hypothesis. It will be helpful to add 2-3 sentences describing how (qualitatively and/or quantitatively) the in vitro-in vivo data correlated based on the demonstrated results.

*Answer: Thank you for the advice. We discussed IVIVC with our coauthors, and discovered we could not perform the IVIVC because this study did not have the immediate release formulation and/or other multiple formulations for testing. This study design was not enough to compare in vivo data to in vitro data. We removed the phrase “in vivo data corresponded to in vitro data”.*

Material and methods

1. Line 125: butterfly ‘valve (is missing)’

*Answer: We revised the description of how we secured the outer tubing to the animal.*

2. Line 153-154: For reproducibility, it will be helpful to mention the value of consistent ratio maintained for the intestinal fluid and quench solution

*Answer: We added the ratio for the intestinal fluid and quenching solution (250:40).*

3. System suitability:

-Need to rephrase this section as the sentences are confusing due to repeated occurrences of the phrase ‘system suitability’.

- For complete definition of method, please include the standard concentration that was selected for these studies.

- What quantitative criteria were set to determine the system suitability/ repeatability?

*Answer: This section has been revised. It now reads: A total 6 replicate samples at low, medium and high concentrations (0.21, 1.05 and 2.8 µM of LA, 0.45, 1.13 and 3 µM of fragment 1, 0.48, 1.2 and 3.2 µM of fragment 2, 0.45, 1.13 and 3 µM of fragment 3 and 0.51, 1.28 and 3.4 µM of fragment 4) were tested on 3 days and interday and intraday precision and accuracy were calculated. Mean of each concentration were within 15 % of the nominal value and have a precision not exceeding 15 % CV (Table S2).*

*We uploaded method suitability study data to the supplementary files.*

Results

1. Reorganize Table 1 to include the volume of intestinal fluid collected from duodenum and jejunum at different time points, respectively and the corresponding LA concentrations

*Answer: We added the volume of intestinal fluid collected from duodenum and jejunum at each time points respectively. In addition, we added the raw data to the supplementary file.*

2. Table 1/Fig. 9: It is unclear how n=3 is obtained, if only 2 pigs were used for the study. Are these biological replicates or technical replicates?

*Answer: We dosed one pig twice with 48 hours washout (n=2). The second pig had a single dose (n=1).*

Discussion and conclusions:

1. The authors need to revise the discussion sections to improve the flow of concepts and connectivity.

For e.g. The statement in line 262 ‘It has been reported…’ fits better in lines 310/311

*Answer: We revised the discussion.*

2. Line 260: The use of adjective ‘meaningful’ is very vague. It is unclear if they want to imply bio-relevance or agreement with qualitative in vivo release profile, etc.

*Answer: We removed this adjective.*

3. While the authors repeatedly state that the in vivo concentrations correlate well with the in vitro dissolution results, there is no explicit description on how these in vitro results were captured, interpreted and (qualitatively) compared/correlated. It will be helpful to provide a detailed discussion on Fig 3 and its implications for Fig 9.

*Answer: See previous explanations on why IVIVC was not performed, which we also included in the revised Discussion.*

Figures

Figure 1 and 2 can be clubbed together

*Answer: Since figure 1 shows amino acid sequence of LA and fragments and figure 2 shows in vivo ultrafiltration probe, we want to keep these figures separately.*

Figure 3:

Is the in vitro dissolution data published in previous literature, or generated for this study? If reusing from previous literature, please cite the source. If the study was performed for this manuscript, include the methodology and result discussion for the same as.

*Answer: We added in vitro methodology, results and discussion.*

Edit axes titles to be more descriptive of the units shown: e.g. X axis- time (minutes), Y-axis- cumulative % dissolved

*Answer: We changed the description of the title of X axis and Y axis.*

It is unclear how samples 1-6 are related to each other. Are they replicates? Please edit the legend appropriately.

*Answer: The six capsules are replicated.*

Figure 5: Elaborate this caption to describe the model and provide legend for notations used (L, S, k, 1, 2 etc.)

*Answer: We added the description of this model and legend for all notations.*

Figure 6: can be clubbed with Fig. 5 or moved to supplementary information

*Answer: We moved figure 6 to the supplementary information.*

Figure 7: Multiple spelling errors

*Answer: We corrected the mistakes such as acclimation and intestinal.*

Figure 8: can be moved to supplementary information

*Answer: We moved figure 8 to the supplementary information.*

Figure 9:

-Edit axes titles to be more descriptive of the units shown: e.g. X axis-time (hr), Y-axis- LA concentration in intestinal fluid (µM)

- (Optional) Can also include a similar dotted line to identify LOD

*Answer: We changed the description of the title of X axis and Y axis and added an LOD line.*

Supplementary information:

(Optional) Include calibration curves and method suitability study data

*Answer: We added calibration curves (S3 figure) and method suitability study data (S2 table) to supplementary information*.

Copyediting recommendations:

- Need to standardize (italicize and/or remove hyphen) the format of words like ‘in vitro’ and ‘in vivo’, ‘et al’ throughout the document.

- Avoid unnecessary capitalization (e.g. line 135: In-vitro, line 461: Pig)

- Maintain a single space between values and their unit of measurements for standardization (e.g. line 29: ‘1 mg’ instead of ‘1mg’) throughout the manuscript

- Line 91: Capitalization of the letter ‘I’ in the word- institutional

- Line 215: Explicitly define ‘CV’ (as coefficient of variation?)

*Answer: All recommendations have been accepted and corrected.*

*CV stands for coefficient of variation.*
